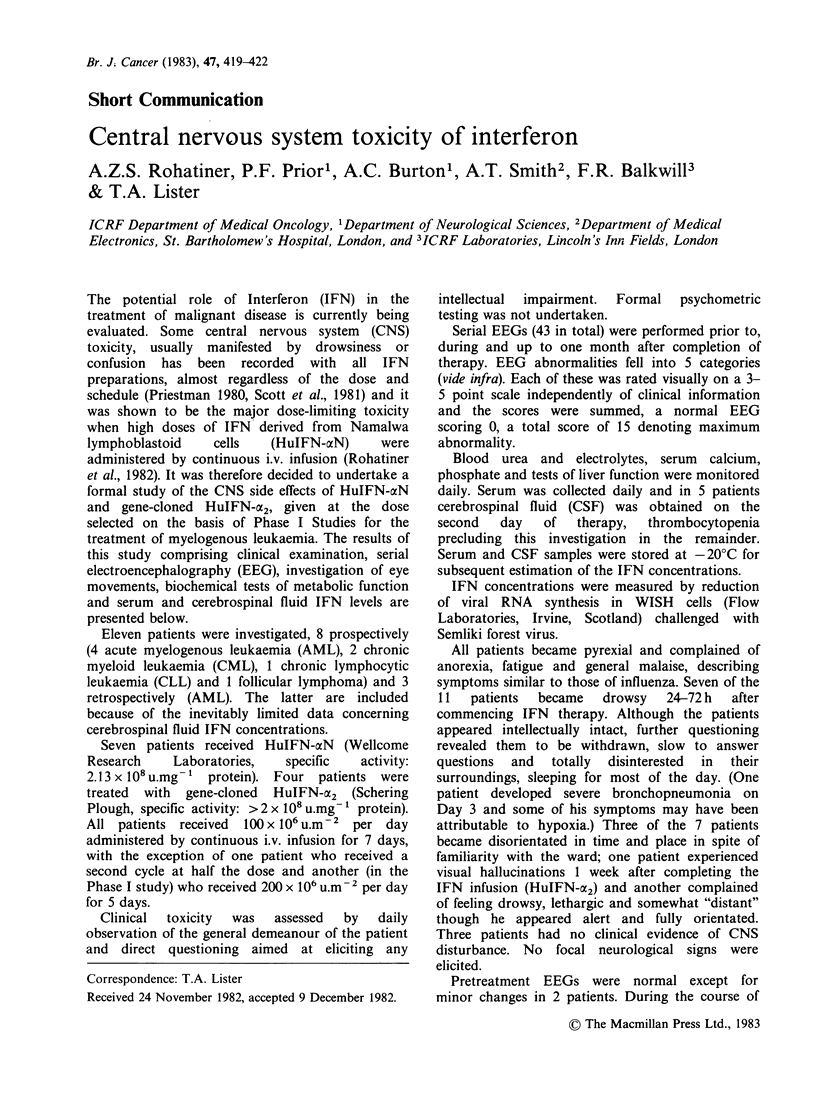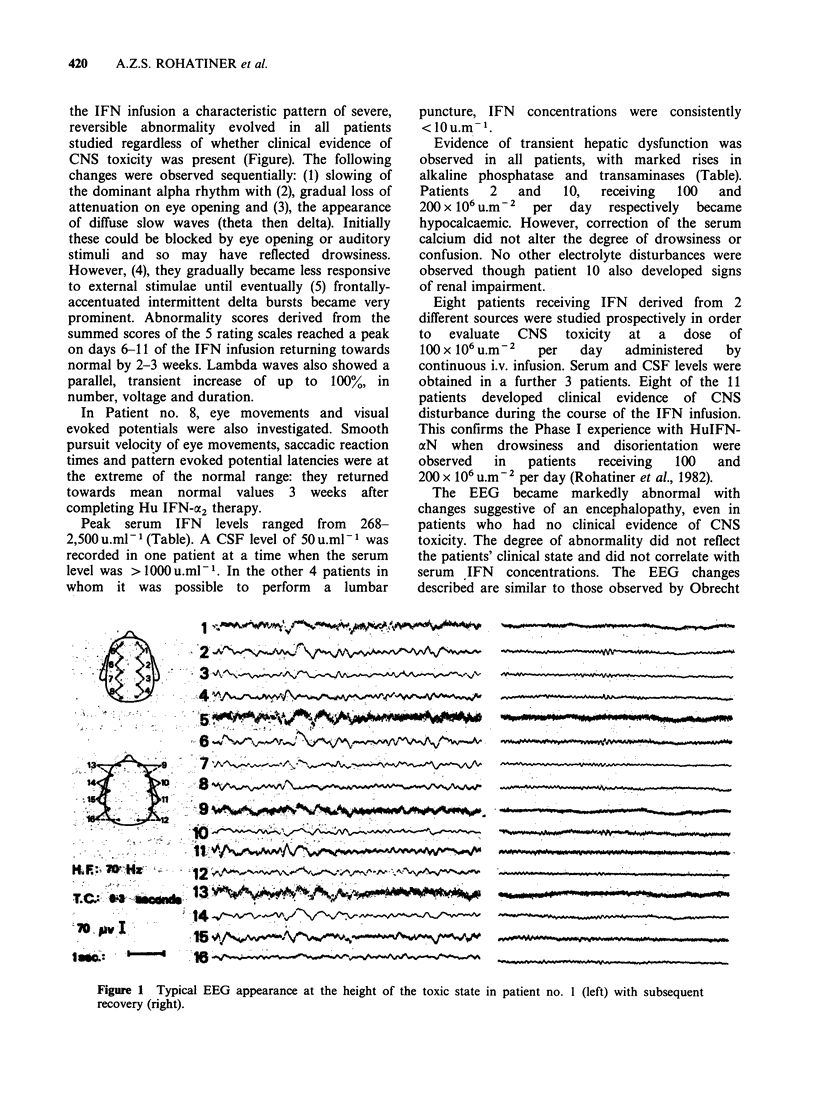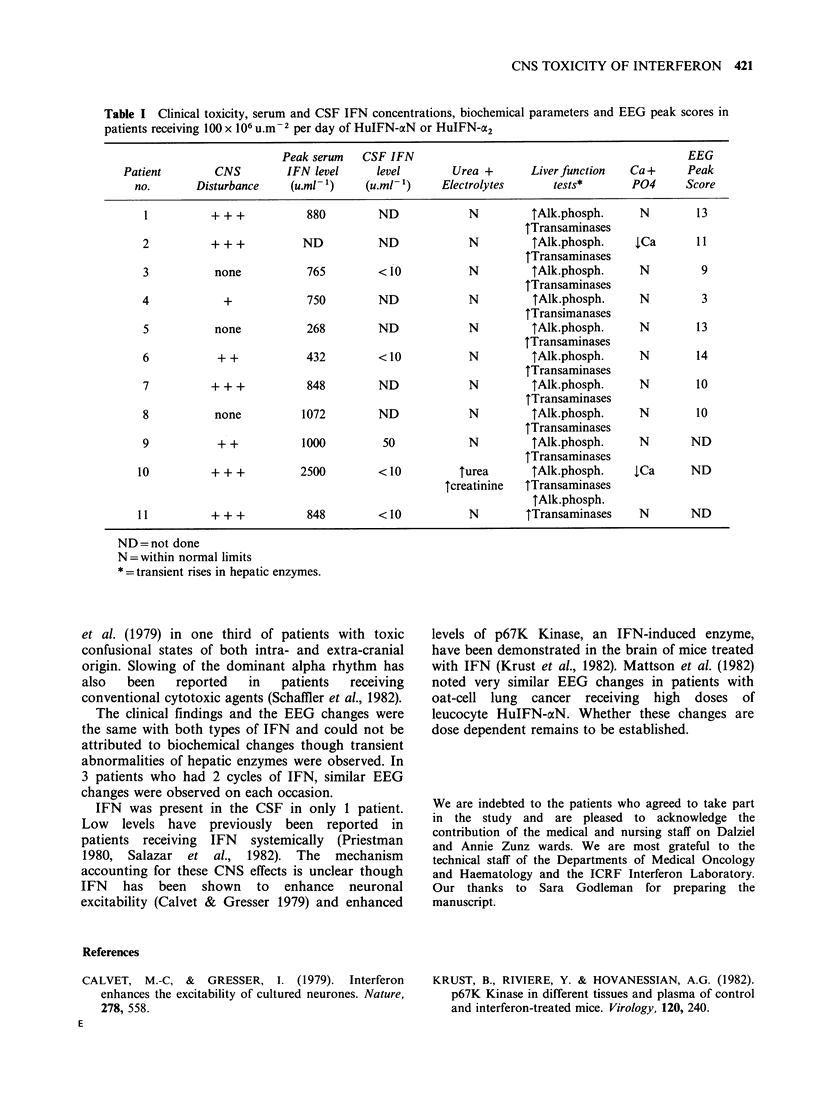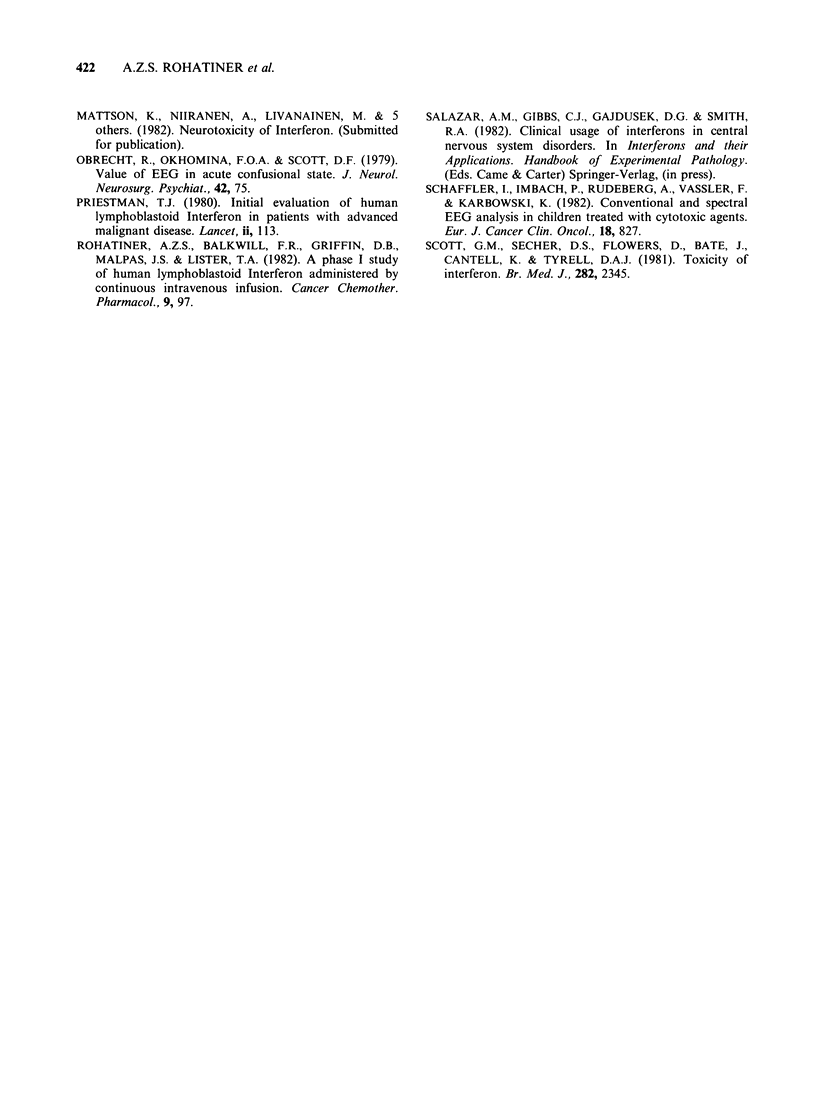# Central nervous system toxicity of interferon.

**DOI:** 10.1038/bjc.1983.63

**Published:** 1983-03

**Authors:** A. Z. Rohatiner, P. F. Prior, A. C. Burton, A. T. Smith, F. R. Balkwill, T. A. Lister


*Br. J. Cancer* (1983), **47**, 419–422

### Short Communication

### Central nervous system toxicity of interferon

A.Z.S. Rohatiner, P.F. Prior[1], A.C. Burton[1], A.T. Smith[2], F.R. Balkwill[3] & T.A. Lister

*ICRF Department of Medical Oncology, [1]Department of Neurological Sciences, [2]Department of Medical Electronics, St. Bartholomew's Hospital, London, and [3]ICRF Laboratories, Lincoln's Inn Fields, London*

The potential role of Interferon (IFN) in the treatment of malignant disease is currently being evaluated. Some central nervous system (CNS) toxicity, usually manifested by drowsiness or confusion has been recorded with all IFN preparations, almost regardless of the dose and schedule (Priestman 1980, Scott *et al.*, 1981) and it was shown to be the major dose-limiting toxicity when high doses of IFN derived from Namalwa lymphoblastoid cells (HuIFN-$\alpha$N) were administered by continuous i.v. infusion (Rohatiner *et al.*, 1982). It was therefore decided to undertake a formal study of the CNS side effects of HuIFN-$\alpha$N and gene-cloned HuIFN-$\alpha_2$, given at the dose selected on the basis of Phase I Studies for the treatment of myelogenous leukaemia. The results of this study comprising clinical examination, serial electroencephalography (EEG), investigation of eye movements, biochemical tests of metabolic function and serum and cerebrospinal fluid IFN levels are presented below.

Eleven patients were investigated, 8 prospectively (4 acute myelogenous leukaemia (AML), 2 chronic myeloid leukaemia (CML), 1 chronic lymphocytic leukaemia (CLL) and 1 follicular lymphoma) and 3 retrospectively (AML). The latter are included because of the inevitably limited data concerning cerebrospinal fluid IFN concentrations.

Seven patients received HuIFN-$\alpha$N (Wellcome Research Laboratories, specific activity: $2.13 \times 10^8$ u.mg$^{-1}$ protein). Four patients were treated with gene-cloned HuIFN-$\alpha_2$ (Schering Plough, specific activity: $>2 \times 10^8$ u.mg$^{-1}$ protein). All patients received $100 \times 10^6$ u.m$^{-2}$ per day administered by continuous i.v. infusion for 7 days, with the exception of one patient who received a second cycle at half the dose and another (in the Phase I study) who received $200 \times 10^6$ u.m$^{-2}$ per day for 5 days.

Clinical toxicity was assessed by daily observation of the general demeanour of the patient and direct questioning aimed at eliciting any intellectual impairment. Formal psychometric testing was not undertaken.

Serial EEGs (43 in total) were performed prior to, during and up to one month after completion of therapy. EEG abnormalities fell into 5 categories (*vide infra*). Each of these was rated visually on a 3–5 point scale independently of clinical information and the scores were summed, a normal EEG scoring 0, a total score of 15 denoting maximum abnormality.

Blood urea and electrolytes, serum calcium, phosphate and tests of liver function were monitored daily. Serum was collected daily and in 5 patients cerebrospinal fluid (CSF) was obtained on the second day of therapy, thrombocytopenia precluding this investigation in the remainder. Serum and CSF samples were stored at $-20$°C for subsequent estimation of the IFN concentrations.

IFN concentrations were measured by reduction of viral RNA synthesis in WISH cells (Flow Laboratories, Irvine, Scotland) challenged with Semliki forest virus.

All patients became pyrexial and complained of anorexia, fatigue and general malaise, describing symptoms similar to those of influenza. Seven of the 11 patients became drowsy 24–72 h after commencing IFN therapy. Although the patients appeared intellectually intact, further questioning revealed them to be withdrawn, slow to answer questions and totally disinterested in their surroundings, sleeping for most of the day. (One patient developed severe bronchopneumonia on Day 3 and some of his symptoms may have been attributable to hypoxia.) Three of the 7 patients became disorientated in time and place in spite of familiarity with the ward; one patient experienced visual hallucinations 1 week after completing the IFN infusion (HuIFN-$\alpha_2$) and another complained of feeling drowsy, lethargic and somewhat "distant" though he appeared alert and fully orientated. Three patients had no clinical evidence of CNS disturbance. No focal neurological signs were elicited.

Pretreatment EEGs were normal except for minor changes in 2 patients. During the course of

Correspondence: T.A. Lister
Received 24 November 1982, accepted 9 December 1982.

© The Macmillan Press Ltd., 1983

the IFN infusion a characteristic pattern of severe, reversible abnormality evolved in all patients studied regardless of whether clinical evidence of CNS toxicity was present (Figure). The following changes were observed sequentially: (1) slowing of the dominant alpha rhythm with (2), gradual loss of attenuation on eye opening and (3), the appearance of diffuse slow waves (theta then delta). Initially these could be blocked by eye opening or auditory stimuli and so may have reflected drowsiness. However, (4), they gradually became less responsive to external stimulae until eventually (5) frontally-accentuated intermittent delta bursts became very prominent. Abnormality scores derived from the summed scores of the 5 rating scales reached a peak on days 6–11 of the IFN infusion returning towards normal by 2–3 weeks. Lambda waves also showed a parallel, transient increase of up to 100%, in number, voltage and duration.

In Patient no. 8, eye movements and visual evoked potentials were also investigated. Smooth pursuit velocity of eye movements, saccadic reaction times and pattern evoked potential latencies were at the extreme of the normal range: they returned towards mean normal values 3 weeks after completing Hu IFN-$\alpha_2$ therapy.

Peak serum IFN levels ranged from 268–2,500 u.ml$^{-1}$ (Table). A CSF level of 50 u.ml$^{-1}$ was recorded in one patient at a time when the serum level was $>1000$ u.ml$^{-1}$. In the other 4 patients in whom it was possible to perform a lumbar puncture, IFN concentrations were consistently $<10$ u.m$^{-1}$.

Evidence of transient hepatic dysfunction was observed in all patients, with marked rises in alkaline phosphatase and transaminases (Table). Patients 2 and 10, receiving 100 and $200\times10^6$ u.m$^{-2}$ per day respectively became hypocalcaemic. However, correction of the serum calcium did not alter the degree of drowsiness or confusion. No other electrolyte disturbances were observed though patient 10 also developed signs of renal impairment.

Eight patients receiving IFN derived from 2 different sources were studied prospectively in order to evaluate CNS toxicity at a dose of $100\times10^6$ u.m$^{-2}$ per day administered by continuous i.v. infusion. Serum and CSF levels were obtained in a further 3 patients. Eight of the 11 patients developed clinical evidence of CNS disturbance during the course of the IFN infusion. This confirms the Phase I experience with HuIFN-αN when drowsiness and disorientation were observed in patients receiving 100 and $200\times10^6$ u.m$^{-2}$ per day (Rohatiner *et al.*, 1982).

The EEG became markedly abnormal with changes suggestive of an encephalopathy, even in patients who had no clinical evidence of CNS toxicity. The degree of abnormality did not reflect the patients' clinical state and did not correlate with serum IFN concentrations. The EEG changes described are similar to those observed by Obrecht

**Figure 1** Typical EEG appearance at the height of the toxic state in patient no. 1 (left) with subsequent recovery (right).

**Table I** Clinical toxicity, serum and CSF IFN concentrations, biochemical parameters and EEG peak scores in patients receiving $100 \times 10^6$ u.m$^{-2}$ per day of HuIFN-αN or HuIFN-$\alpha_2$

| *Patient no.* | *CNS Disturbance* | *Peak serum IFN level (u.ml$^{-1}$)* | *CSF IFN level (u.ml$^{-1}$)* | *Urea + Electrolytes* | *Liver function tests** | *Ca+ PO4* | *EEG Peak Score* |
|---|---|---|---|---|---|---|---|
| 1 | + + + | 880 | ND | N | ↑Alk.phosph. ↑Transaminases | N | 13 |
| 2 | + + + | ND | ND | N | ↑Alk.phosph. ↑Transaminases | ↓Ca | 11 |
| 3 | none | 765 | < 10 | N | ↑Alk.phosph. ↑Transaminases | N | 9 |
| 4 | + | 750 | ND | N | ↑Alk.phosph. ↑Transimanases | N | 3 |
| 5 | none | 268 | ND | N | ↑Alk.phosph. ↑Transaminases | N | 13 |
| 6 | + + | 432 | < 10 | N | ↑Alk.phosph. ↑Transaminases | N | 14 |
| 7 | + + + | 848 | ND | N | ↑Alk.phosph. ↑Transaminases | N | 10 |
| 8 | none | 1072 | ND | N | ↑Alk.phosph. ↑Transaminases | N | 10 |
| 9 | + + | 1000 | 50 | N | ↑Alk.phosph. ↑Transaminases | N | ND |
| 10 | + + + | 2500 | < 10 | ↑urea ↑creatinine | ↑Alk.phosph. ↑Transaminases | ↓Ca | ND |
| 11 | + + + | 848 | < 10 | N | ↑Alk.phosph. ↑Transaminases | N | ND |

ND = not done
N = within normal limits
* = transient rises in hepatic enzymes.

*et al.* (1979) in one third of patients with toxic confusional states of both intra- and extra-cranial origin. Slowing of the dominant alpha rhythm has also been reported in patients receiving conventional cytotoxic agents (Schaffler *et al.*, 1982).

The clinical findings and the EEG changes were the same with both types of IFN and could not be attributed to biochemical changes though transient abnormalities of hepatic enzymes were observed. In 3 patients who had 2 cycles of IFN, similar EEG changes were observed on each occasion.

IFN was present in the CSF in only 1 patient. Low levels have previously been reported in patients receiving IFN systemically (Priestman 1980, Salazar *et al.*, 1982). The mechanism accounting for these CNS effects is unclear though IFN has been shown to enhance neuronal excitability (Calvet & Gresser 1979) and enhanced levels of p67K Kinase, an IFN-induced enzyme, have been demonstrated in the brain of mice treated with IFN (Krust *et al.*, 1982). Mattson *et al.* (1982) noted very similar EEG changes in patients with oat-cell lung cancer receiving high doses of leucocyte HuIFN-αN. Whether these changes are dose dependent remains to be established.

We are indebted to the patients who agreed to take part in the study and are pleased to acknowledge the contribution of the medical and nursing staff on Dalziel and Annie Zunz wards. We are most grateful to the technical staff of the Departments of Medical Oncology and Haematology and the ICRF Interferon Laboratory. Our thanks to Sara Godleman for preparing the manuscript.

### References

CALVET, M.-C, & GRESSER, I. (1979). Interferon enhances the excitability of cultured neurones. *Nature*, **278**, 558.

KRUST, B., RIVIERE, Y. & HOVANESSIAN, A.G. (1982). p67K Kinase in different tissues and plasma of control and interferon-treated mice. *Virology*, **120**, 240.

MATTSON, K., NIIRANEN, A., LIVANAINEN, M. & 5 others. (1982). Neurotoxicity of Interferon. (Submitted for publication).

OBRECHT, R., OKHOMINA, F.O.A. & SCOTT, D.F. (1979). Value of EEG in acute confusional state. *J. Neurol. Neurosurg. Psychiat.*, **42,** 75.

PRIESTMAN, T.J. (1980). Initial evaluation of human lymphoblastoid Interferon in patients with advanced malignant disease. *Lancet*, **ii,** 113.

ROHATINER, A.Z.S., BALKWILL, F.R., GRIFFIN, D.B., MALPAS, J.S. & LISTER, T.A. (1982). A phase I study of human lymphoblastoid Interferon administered by continuous intravenous infusion. *Cancer Chemother. Pharmacol.*, **9,** 97.

SALAZAR, A.M., GIBBS, C.J., GAJDUSEK, D.G. & SMITH, R.A. (1982). Clinical usage of interferons in central nervous system disorders. In *Interferons and their Applications. Handbook of Experimental Pathology.* (Eds. Came & Carter) Springer-Verlag, (in press).

SCHAFFLER, I., IMBACH, P., RUDEBERG, A., VASSLER, F. & KARBOWSKI, K. (1982). Conventional and spectral EEG analysis in children treated with cytotoxic agents. *Eur. J. Cancer Clin. Oncol.*, **18,** 827.

SCOTT, G.M., SECHER, D.S., FLOWERS, D., BATE, J., CANTELL, K. & TYRELL, D.A.J. (1981). Toxicity of interferon. *Br. Med. J.*, **282,** 2345.